# Diversity of flora used for the cure of equine diseases in selected peri-urban areas of Punjab, Pakistan

**DOI:** 10.1186/1746-4269-9-70

**Published:** 2013-09-30

**Authors:** Khurram Goraya, Zafar Iqbal, Muhammad Sohail Sajid, Ghulam Muhammad, Qurat ul Ain, Muhammad Saleem

**Affiliations:** 1 Department of Parasitology, University of Agriculture, Faisalabad 38040 Punjab, Pakistan; 2 Department of Veterinary Clinical Medicine and Surgery, University of Agriculture, Faisalabad 38040 Punjab, Pakistan; 3 Brookes Hospital for Animals, Faculty of Veterinary Science, University of Agriculture, Faisalabad 38040 Punjab, Pakistan

**Keywords:** Phytotherapy, Plants, Equines, Indigenous, Ethnobotanicals, Punjab, Pakistan

## Abstract

**Background:**

Plants have widely been used and documented for their therapeutic potential in many parts of the world. There are, however, few reports on the use of plants for the treatment of diseases of equines. To this end, participatory epidemiology and rapid rural appraisal techniques were used to document the plants having pharmacotherapeutic significance against different ailments of equines in selected population of Punjab, Pakistan.

**Methods:**

A survey was conducted to interview a total of 450 respondents (150 from each of the districts of Faisalabad, Lahore and Sargodha of Pakistan) to collect information about disease recognition of the equines and their treatment on a well − structured questionnaire. A total of 60 plants belonging to 40 families were documented. An inventory was developed depicting detailed information of plants used in treatment of different conditions of equines.

**Results:**

The top ten species of plants used were: *Allium cepa, Zingiber officinale, Vernonia anthelmintica, Capsicum annum, Brassica campestris, Trachyspermum ammi, Anethum graveolens, Picrorhiza kurroa, Azadirachta indica,* and *Citrullus colocynthis*. Seeds were the most frequently used (n = 16/60) parts, followed by leaves (n = 12/60) and fruits (n = 11/60) of plants. Based on the combination of different parts of plants used in different ratios and variation in their dose or mode of preparation led to a large number of recipes/remedies against wounds, lameness, bronchitis, colic, anorexia, dermatitis, weakness, parasitism (internal & external), fever, heat stress, urine retention, swelling, toxemia, and indigestion.

**Conclusions:**

This study generated lot of data on phytomedicinal approach for the treatment of ailments in the equines in some selected areas. It would, therefore, be imperative to expand similar studies in other parts of Pakistan and elsewhere. Moreover, use of the documented plants may be validated employing standard scientific procedures, which may have their application in the drug discovery/development by the pharmaceutical industry.

## Background

Equines (horses, donkeys and mules) are playing key roles in providing an economical draught power to resource-poor countries like Pakistan. Equines suffer from a variety of health conditions that not only hamper optimum performance, but also cause huge losses due to mortality [1]. Parasitism has been reported as the major health issue of equines in Punjab, Pakistan followed by wound, bacterial infections, lameness, bronchitis, dermatitis, and colic [2]. In addition to allopathic/modern medicine, there is extensive use of traditional herbs for the treatment of different diseases in equines all over the world [3]. Ethnobotany has revealed that the indigenous knowledge of a community is a key player in the identification of medicinal plants which have been tested through generations in the human history [4]. Traditional medicine and bio-prospecting [5] may often lead to the development of a new herbal product based on their use by significant numbers of people over the extended periods of time [6]. The plant-based medicines have particularly been found promising as anti-parasitics, stomachics, and in treatment of various respiratory ailments [7]–[12]. So far, only a handful of investigations are reported on the use of plants for different ailments/conditions; however, inventories of plants for phyotherapy in the food animals are extensively reported. The present study was, therefore, carried out to document the plants being used in traditional veterinary practices for equines in some selected peri-urban areas of Punjab (Pakistan) where equines are frequently used for different purposes.

## Methods

### Study districts

**Figure 1:**
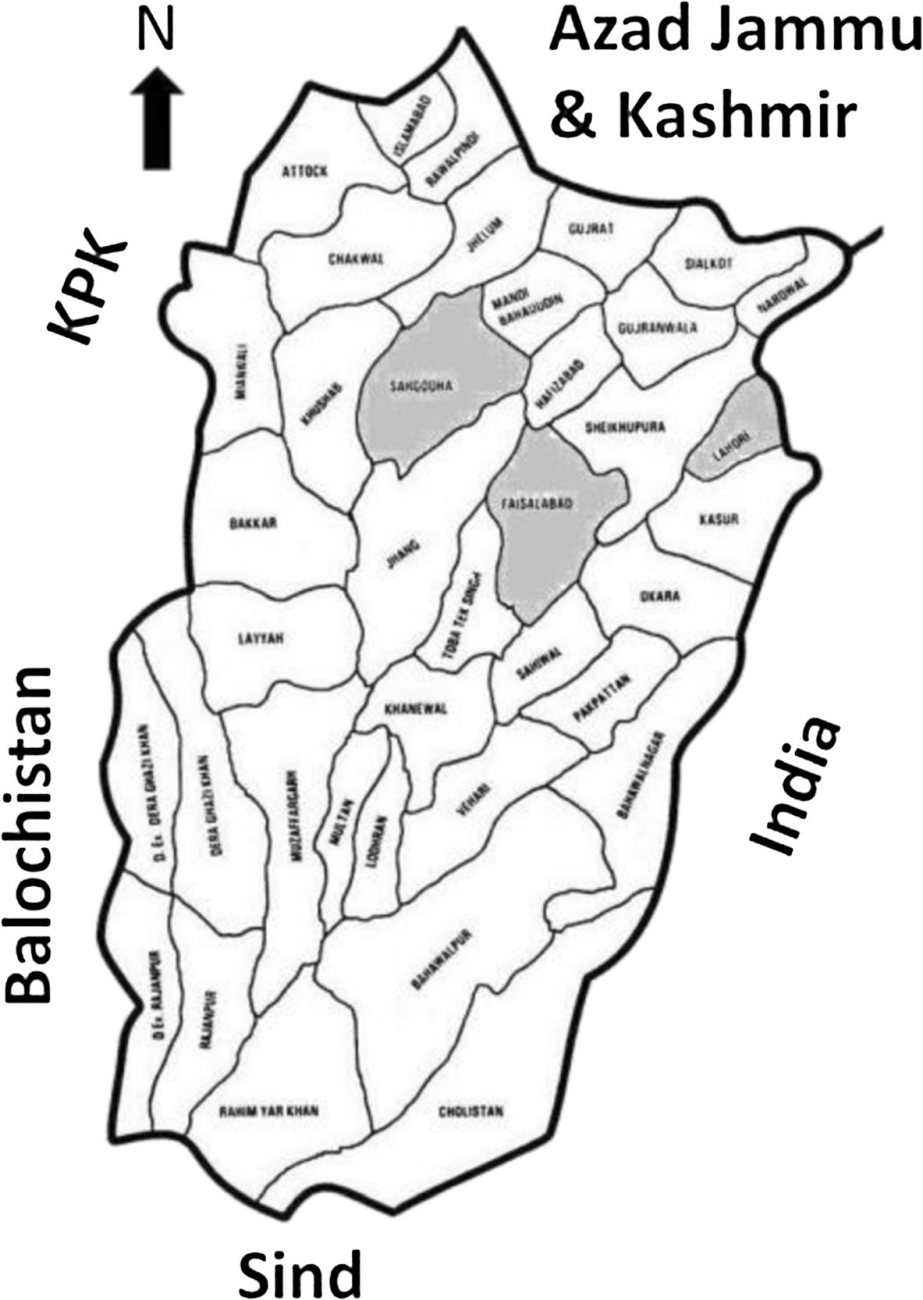
Physical map of the Punjab province, Pakistan indicating districts Faisalabad, Sargodha and Lahore.

Three districts of the central Punjab; Faisalabad, Lahore and Sargodha were included in the present survey. District Lahore is the capital of Punjab (second largest city of Pakistan after Karachi), while district Faisalabad is the hub of textiles (third largest city) of the country. District Sargodha is comparatively smaller city and considered as an agricultural trade center with various industries. The equine population of the three districts has been estimated as 24628 horses, 174994 donkeys and 7849 mules [13]. The use of equines in the three selected industrial districts of Punjab is frequent because it is the cheapest source for carriage of industrial raw materials and products from and to the market [2]. Figure 1 shows physical map of Punjab province and the three study districts.

### Selection of respondents

A small scaled rapid rural appraisal (RRA), an exploratory phase [14] was conducted in metropolitan Faisalabad, Punjab, Pakistan for the purpose of collecting an initial data from candidates who could participate in the second (surveillance) phase of the project.

Of the total 1000 registered farmers (who submitted their willingness to participate in the survey), 450 were selected as key respondents for this study. One hundred and fifty respondents represented each of the three districts of Faisalabad, Lahore and Sargodha. Selection of respondents within each district was done using proportional allocation and map grid methods in order to collect information from the selected districts. The selected respondents belonged to 9 sites each of districts Faisalabad and Sargodha, and 6 sites of Lahore (Table 1).

**Table 1:** List of district wise sites for selection of respondents

**Districts/sites**	**1**	**2**	**3**	**4**	**5**	**6**	**7**	**8**	**9**
Faisalabad	Sidhupura	Chak 79	Ahmad Nagar	Pansera	Chakera	Naitheri wala	Aziz Town	Karad Wala	Brooke Hospital for Animal Static Clinic (UAF)
Sargodha	Noor colony	Chak 87	Chak 88 South	Chak 34	Fatima Jinnah Road	Farooq Colony	Makam-e-Hayat	Chak 88 North	Saido wanan
Lahore	Shahdra Town	Fazal Park	Raiwand	Badian	Sharakpur	Thokar Niaz Baig			

### Participatory epidemiology and collection of data

A questionnaire containing a blend of open ended and closed (dichotomous and multiple choice) questions was prepared and refined through formal and informal testing [15]. For effective communication and data recording, a survey team was appointed; comprising of a veterinarian, professionals of The Brooke Hospital for Animals, and a community leader from the local village. Interviews, focused group discussions, and field visits were conducted with the respondents. The information about disease recognition of the equines and their treatments was collected using the well-structured questionnaire, open-ended interviews and guided-dialogue techniques. Focused group discussions were arranged to cross-check/verify the information provided by the respondents to reach more accurate results. The respondents were asked to tell how they acquired the knowledge of phytotherapy related to the disease/condition recognition of equines. In addition, the direct observation approach was also followed as described by Etkins [16]. Thus, local names of plants, dose, parts of plant used, methods of preparation, and mode of administration were recorded. The farmers were asked to show the plant species described for the treatment of diseases/conditions for their taxonomic identification by the botanists at the Department of Botany, University of Agriculture, Faisalabad, Pakistan and the voucher specimens were preserved for record. The information collected was maintained in Microsoft Excel for further analyses and interpretation.

## Results

### Respondents

Equine owners/traditional veterinary healers were well familiar with the signs and symptoms of the diseases/conditions of equines in the study area and a majority of the botanical ingredients used in treatment were of indigenous origin. Therefore, farmers had these remedies available at their door step or at the most in the nearby grocery shops. The respondents were not trained by any authority about usage of plants for treatment of their animals. In fact, their knowledge was based on folk beliefs and previous practices based on hit and trial methods.

### Diversity of plants used

A total of 60 plants were documented (Table 2) for their use in different diseases/conditions of equines. Documented plants represented 40 families. Fabaceae was the largest represented family including five plants, followed by other families (Table 2). Composition, dosage, mode of preparation and administration, and frequency of usage of Traditional Veterinary Practices (TVPs) has been presented in Table 3. Plants and diversity of their usage in different diseases have been presented in Table 4. Thirty one species were used for the treatment of multiple diseases/conditions. Piyaz (Onion; *Allium cepa*)*,* Adrak (Ginger; *Zingiber officinale*)*,* Kali zeeri (Iron weed; *Vernonia anthelmintica*)*,* Mirch (Chilli; *Capsicum annum*)*,* Sarsoon (Rapeseed plany; *Brassica campestris*)*,* Ajwain (Carom seeds; *Trachyspermum ammi*)*,* Ajwain (Dill; *Anethum graveolens*)*,* Kutka (*Picrorhiza kurroa*)*,* Neem (*Azadirachta indica*), and Kor tumma (Bitter gourd; *Citrullus colocynthis*) were the top ten most frequently used plants as part of prescriptions in different diseases/conditions of equines.

**Table 2:** An inventory of floral diversity documented by local respondents (n=450) from three districts (Faisalabad, Sargodha and Lahore) of Punjab, Pakistan for the treatment of equine disease

**Sr.**	**Botanical name/**	**Local name**	**Family**	**Part (s) used**	**Usage diversity**	**No. of**	**Part of**
**No.**	**English name**					**prescriptions**	**remedies**
1.	*Acacia nilotica* (L.) Willd. ex Delile	Desi kikar	Fabaceae	Branches	Internal parasites, swelling	2	2
2.	*Allium cepa* L. var. *aggregatum* G. Don	Piyaz	Liliaceae	*Bulb*	Anorexia, bad habits, bronchitis, colic, diarrhea, fever, heat stress, indigestion, pain, quidding, weakness	11	33
3.	*Allium sativum* L.	Lehsan	Liliaceae	Bulb	Bronchitis, fever, indigestion	3	3
4.	*Aloe vera* (L.) Burm. f.	Kawar gandal	Aloaceae	*Leaves*	Anorexia, bronchitis	2	2
5.	*Amomum subulatum* Roxb.	Ilaichi	Zingiberaceae	Fruit	Fever	1	1
6.	*Anethum graveolens* L.	Soye	Umbelliferae	Seeds	Anorexia, bronchitis, colic, fever, indigestion, lameness, toxemia, weakness	8	10
7.	*Azadirachta indica* A. Juss*.*	Neem	Meliaceae	Leaves	Dermatitis, external parasites, lameness, wound	5	13
8.	*Bambusa bambos* L.	Bans	Bambusaceae	Leaves	Bronchitis	1	1
9.	*Brassica campestris* L. ssp. *napus* Duthie and Fuller	Surson	Brassicaseae	*Seeds, seed oil*	Colic, dermatitis, diarrhea, external parasites, lameness, retention of urine, swelling, weakness, wound	8	47
10.	*Calotropis procera* (Ait.) W.T.Ait.	Aak	Asclepiadaceae	Buds	Weakness	1	1
11.	*Capsicum annum* L.	Subz mirch	Solanaceae	Fruit	Anorexia, bronchitis, fever, indigestion, lameness, quidding, retention of urine, toxemia, weakness	9	19
12.	*Cascuta reflexa* Roxb.	Akas bail	Cuscutaceae	Whole plant	Lameness	1	1
13.	*Cicer arietinum* L.	Kalay chaney	Fabaceae	Seeds	Dermatitis, lameness, wound	3	4
14.	*Citrullus colocynthis* (L*.*) Schrad.	Kor tumma	Cucurbitaceae	Fruit	Anorexia, fever, indigestion, weakness	4	9
15.	*Citrus limon* (L.) Burm.	Nimbu	Rutaceae	Fruit	Lameness, wound	2	2
16.	*Cocos nucifera* L.	Giri	Arecaceae	Oil	Lameness	1	1
17.	*Curcuma longa* L.	Haldi	Zingiberaceae	Rhizome	External parasite, lameness, wound	3	8
18.	*Eruca sativa* Mill.	Tara mera	Cruciferae	Seed, seed oil	Dermatitis, external parasite, wound	3	5
19.	*Eugenia caryophyllata* Thunb.	Loung	Myrtaceae	Fruit	Anorexia, lameness, pain	3	4
20.	*Euphorbia caducifolia* Haines.	Danda thor	Euphorbiaceae	Branches	Colic	1	1
21.	*Ficus religiosa* L.	Pipal	Moraceae	Bark	Swelling	1	1
22.	*Foeniculum vulgare* P. Mill.	Sounf	Apiaceae	Seeds	Indigestion, weakness	2	2
23.	*Geranium wallichianum* D. Don Ex Sweet	Ratan jot	Geraniaceae	Seeds	Lameness, wound	2	4
24.	*Glycyrrhiza glabra* L.	Mullathi	Fabaceae	*Roots*	Bronchitis	1	7
25.	*Grewia asiatica* L.	Falsa	Tiliaceae	Fruit	Lameness	1	1
26.	*Halorrhena pubescens* Wall. ExG. Don.	Kuro	Apocynaceae	Bark	Heat stress	1	1
27.	*Hordeum vulgare* L.	Jow	Poaceae	Seeds	Heat stress	1	1
28.	*Lagenaria siceraria* Molina	Kuddo	Cucurbitaceae	Leaves	Internal parasite	1	1
29.	*Lawsonia inermis* L.	Mehndi	Lythraceae	Leaves	Wound	1	7
30.	*Lens culinaris* Medik.	Masoor	Fabaceae	*Seeds*	Wound	1	1
31.	*Lepidium sativum* L.	Halion	Apiaceae	Seeds	Internal parasites, weakness	2	2
32.	*Linum usitatissimum* L.	Alsi	Linaceae	Seeds	Internal parasites	1	1
33.	*Mallotus philippinensis* (Lamk.) Meull. Arg.	Kamela	Euphorbiaceae	Fruit	Anorexia, internal parasites	2	4
34.	*Mangifera indica* L.	Aam	Anacardiaceae	Fruit	Lameness	1	1
35.	*Medicago sativa* L.	Lusan	Papilionaceae	Leaves	Lameness	1	1
36.	*Mentha longifolia* (L.) Huds.	Podina	Lamiaceae	Leaves	Anorexia	1	1
37.	*Myristica fragrans* HOUTT.	Jaful	Myristicaceae	Fruit	Lameness, pain, tetanus	3	5
38.	*Nicotiana tabacum* L.	Tambaku	Solanaceae	Leaves	Colic, pain	2	3
39.	*Nigella sativa* L.	Kalonji	Ranunculaceae	Seeds	Anorexia, bronchitis	2	2
40.	*Olea europaea* L.	Zaytoon	Oleaceae	Fruit	Lameness	1	1
41.	*Oryza sativa* L.	Chawal	Poaceae	*Whole plant*	Internal parasites	1	1
42.	*Peganum harmala* L.	Hurmil	Zygophyllaceae	Fruit	Anorexia, lameness, pain, swelling	4	4
43.	*Pennisetum glaucum* L.	Bajra	Poaceae	Whole	Lameness	1	1
44.	*Picrorhiza kurroa* Royle ex. Benth.	Kourdh	Scrofulariaceae	Rhizome	Anorexia, fever, indigestion, lameness, tetanus, weakness	6	11
45.	*Piper betle* L.	Paan	Piperaceae	Leaves	Anorexia	1	1
46.	*Piper nigrum* L.	Kali mirch	Piperaceae	Pepper corn	Anorexia, bronchitis, toxemia	3	5
47.	*Prunus dulcis* Mill.	Badam	Rosaceae	Seed	Diarrhea	1	1
48.	*Ricinus communis* L.	Arind	Euphorbiaceae	Flower oil	Internal parasites	1	1
49.	Rosa damascena Mill.	Gulab	Rosaceae	Flowers	Bronchitis	1	4
50.	*Sesamum indicum* L.	Til	Pedaliaceae	Seeds, seed oil	Colic, dermatitis, lameness, pain	4	7
51.	*Trachyspermum ammi* (L.) Sprague ex Turrill.	Ajwain	Apiaceae	*Seeds*	Anorexia, bronchitis, colic, fever, heat stress, indigestion, lameness, retention of urine	8	19
52.	*Trifolium alexandrinum* L.	Barseem	Papilionaceae	Whole plant	Wound	1	1
53.	*Trigonella foenum-graecum* L.	Methray	Fabaceae	Seeds	Toxemia	1	1
54.	*Triticum aestivum* L.	Gundum	Poaceae	*Flour, grain*	External parasites, lameness, weakness, wound	4	5
55.	*Vernonia anthelmintica* (L*.*) Willd.	Kali zeeri	Asteraceae	Seeds	Anorexia, bronchitis, colic, diarrhea, heat stress, indigestion, lameness, toxemia, + Weakness+ Wound	10	23
56.	*Withania coagulans* Dunal	Paneer	Solanaceae	Leaves	Anorexia, fever, indigestion, weakness	4	4
57.	*Withania somnifera* L. Dunal	Aksan	Solanaceae	Leaves	Wound	1	1
58.	*Zea mays* L.	Makai	Gramineae	Flour	Anorexia, haematuria, weakness, wound	4	5
59.	*Zingiber officinale* Roscoe	Adrak	Zingiberaceae	Rhizome	Anorexia, bronchitis, colic, fever, heat stress, indigestion, lameness, pain, tetanus, weakness	10	26
60.	*Ziziphus jujuba* L. Lam., non P. Mill.	Beri	Rhamnaceae	Leaves	Wound	1	1

**Table 3:** List of traditional veterinary practices based on plant materials for the treatment of different diseases/conditions of equines reported by the local respondents (n=450) in the study area

**S.**	**Phytotherapeutic material used**	**Dose and administration**	**Usage**
**No.**			
	**Anorexia**		
1	*Zingiber officinale* (rhizome) + *Capsicum annum* (fruit) + *Allium cepa* (bulb) + Common salt	50 g + 125 g + 250 g + 125 g; MGB/PO (Mix, Grind, and make a Bolus to administer Per Oss)	2
2	*Allium cepa* (bulb) + *Citrullus colocynthis* (fruit) + *Capsicum annum* (fruit) + *Zingiber officinale* (rhizome) + Jaggery	125 g + 50 g + 125 g + 50 g + 100 g; MGB/PO	1
3	*Allium cepa* (bulb) + *Zingiber officinale* (rhizome) + Black salt	250 g + 100 g + 100 g; MGB/PO - 2-3 days	1
4	*Capsicum annum* (fruit) + *Allium cepa* (bulb) + *Vernonia anthelmintica* (seeds) + *Eugenia caryophyllata* (fruit) + *Zingiber officinale* (rhizome) + Common salt	500 g + 250 g + 250 g + 100 g + 250 g + 250 g; MGB/PO	1
5	*Capsicum annum* (fruit) + *Allium cepa* (bulb) + *Zingiber officinale* (rhizome) + *Trachyspermum ammi* (seeds) + *Nigella sativa* (seeds) + Black salt + Common salt + *Picrorhiza kurroa* (rhizome) + *Citrullus colocynthis* (fruit) + *Peganum harmala* (fruit)	125 g + 125 g + 50 g + 25 g + 50 g + 50 g + 50 g + 50 g + 50 g; MGB/PO – divide into 02 doses for 02 days	1
6	Common salt + *Allium cepa* (bulb) + *Capsicum annum* (fruit)	125 g + 250 g + 125 g; MGB/PO	1
7	Common salt + Black salt + *Allium cepa* (bulb) + *Piper nigrum* (pepper corn) + *Vernonia anthelmintica* (seeds) + *Trachyspermum ammi* (seeds)	50 g + 50 g + 50 g + 25 g + 25 g + 25 g; MGB/PO	1
8	*Zea mays* (flour) + Brown sugar	250 g + 250 g; MGB/PO	1
9	*Zea mays* (flour) + Brown sugar + Water	250 g + 250 g + 5 L; Mix and give PO	1
10	Jaggery + *Allium cepa* (bulb)	250 g + 500 g; MGB/PO	1
11	*Mallotus philippinensis* (fruit) + Yogurt	10 g + 125 g; MGB/PO	1
12	*Mentha longifolia* (leaves) + *Capsicum annum* (fruit) + Common salt + *Allium cepa* (bulb)	125 g + 50 g + 50 g + 125 g; MGB/PO	1
13	*Picrorhiza kurroa* (rhizome) + *Citrullus colocynthis* (fruit) + *Aloe vera* (leaves) + *Allium cepa* (bulb)	125 g + 125 g + 50 g; MGB/PO	1
14	*Piper nigrum* (pepper corn) + Black salt + *Vernonia anthelmintica* (seeds) + *Capsicum annum* (fruit)	50 g + 25 g + 25 g + 25 g; MGB/PO	1
15	*Trachyspermum ammi* (seeds) + *Vernonia anthelmintica* (seeds) + Jaggery	25 g + 50 g + 125 g; MGB/PO	1
16	*Trachyspermum ammi* (seeds) + *Zingiber officinale* (rhizome) + *Piper nigrum* (pepper corn) + *Piper betle* (leaves) + Jaggery	50 g + 50 g + 50 g + 25 g + 250 g; MGB/PO	1
17	*Vernonia anthelmintica* (seeds) + *Anethum graveolens* (seeds) + Black salt + *Zingiber officinale* (rhizome) + *Withania coagulans* (leaves) + Common salt + *Picrorhiza kurroa* (rhizome) + *Citrullus colocynthis* (fruit) + Jaggery + *Allium cepa* (bulb) + *Capsicum annum* (fruit)	125 g + 125 g + 12 g + 50 g + 250 g + 50 g + 250 g + 500 g + 250 g + 250 g + 250 g; MGB/PO	1
18	*Zingiber officinale* (rhizome) + *Allium cepa* (bulb) + *Capsicum annum* (fruit) + *Vernonia anthelmintica* (seeds) + Black salt	125 g + 1 kg + 250 g + 50 g + 250 g; MGB/PO	1
	**Total entries**		**19**
	**Bad habit (Mud eating)**		
1	Jaggery + *Allium cepa* (bulb)	250 g + 250 g; Boiled bulbs mixed with jiggery – given PO	1
	**Total entries**		**1**
	**Bronchitis**		
1	*Glycyrrhiza glabra* (roots) + Jaggery	250 g + 250 g; MGB/PO – 2-3 days	4
2	Jaggery + *Allium cepa* (bulb)	250 g + 250 g; MGB/PO – 2-3 days	4
**3**	Nuswar	10 g nuswar pushed in nose with a pipe	3
4	*Glycyrrhiza glabra* (roots) + *Rosa damascena* (flowers) + *Piper nigrum* (pepper corn)	250 g + 375 g + 25 g; MGB/PO – 2-3 days	2
5	*Glycyrrhriza glabra* (roots) + *Zingiber officinale* (rhizome) + *Allium cepa* (bulb) + Jaggery	50 g + 50 g + 125 g + 125 g; MGB/PO	2
7	*Bumbusa bambos* (leaves)	500 g PO	1
8	*Glycyrrhiza glabra* (roots) + *Allium cepa* (bulb) + *Nigella sativa* (seeds) + Ammonium chloride + Jaggery	250 g + 2 kg + 250 g + 125 g + 2 kg; Mixed, ground and fried to make custard – 125 g daily for 5 days	1
9	*Glycyrrhriza glabra* (roots) + Jaggery + *Allium cepa* (bulb)	50 g + 250 g + 250 g; MGB/PO	1
10	*Glycyrrhiza glabra* (roots) + Jaggery + *Vernonia anthelmintica* (seeds) + *Anethum graveolens* (seeds) + *Allium cepa* (bulb)	25 g + 250 g + 25 g + 25 g + 250 g; MGB/PO	1
11	*Glycyrrhiza glabra* (roots) + *Rosa damascena* (flowers) + *Allium cepa* (bulb)	125 g + 250 g + 500 g; MGB/PO	1
12	*Glycyrrhiza glabra* (roots) + *Rosa damascena* (flowers) + Ammonium chloride + *Piper nigrum* (pepper corn) + *Allium cepa* (bulb) + Jaggery + Water	125 g + 125 g + 25 g + 25 g + 250 g + 125 g + 125 ml; Mixed, fried and made custard – given PO	1
13	*Glycyrrhiza glabra* (roots) + *Rosa damascena* (flowers) + *Zingiber officinale* (rhizome)	125 + 125 g + 125 g; MGB/PO	1
14	*Aloe vera* (leaves) + *Capsicum annum* (fruit) + *Zingiber officinale* (rhizome) + *Allium sativum* (bulb) + *Vernonia anthelmintica* (seeds)	125 g + 125 g + 50 g + 50 g + 50 g; MGB/PO	1
15	Jaggery + *Allium cepa* (bulb) + *Glycyrrhiza glabra* (roots) + *Zingiber officinale* (rhizome) + *Piper nigrum* (pepper corn)	250 g + 500 g + 100 g + 100 g + 50 g; MGB/PO	1
16	Joshanda + Jaggery + *Allium cepa* (bulb) + Water	1 kg + 1 kg + 2 kg + 2 L; Mixed, boiled in water till concentrated – given PO	1
17	*Rosa damascena* (flowers) + *Glycyrrhiza glabra* (roots) + Ammonium chloride	375 g + 50 g + 10 g; MGB/PO	1
18	*Trachyspermum ammi* (seeds) + Jaggery + Common salt	100 g + 200 g + 50 g; MGB/PO	1
19	*Trachyspermum ammi* (seeds) + *Vernonia anthelmintica* (seeds) + Jaggery	25 g + 25 g + 125 g; MGB/PO	1
	**Total entries**		**28**
	**Colic**		
1	*Brassica campestris* (seed oil)	500 ml – PO	2
2	*Brassica campestris* (seed oil) + Water	250 ml + 250 ml – PO	2
3	*Nicotiana tabacum* (leaves) + Jaggery	50 g + 250 g; MGB/PO	2
6	*Allium cepa* (bulb)	500 g juice – PO	1
7	Ammonium chloride + *Brassica campestris* (seed oil) + Common salt	50 g + 125 ml + 50 g; Mix and give PO	1
8	Ammonium chloride + Potassium bicarbonate	50 g + 50 g; MGB/PO	1
9	*Euphorbia caducifolia (branches)* + Water	10 ml juice mixed in 250 ml water – PO	1
11	Jaggery + *Allium cepa* (bulb)	250 g + 250 g; MGB/PO	1
12	Jaggery + *Nicotiana tabacum* (leaves)	250 g + 50 g; Mix and give PO	1
13	Sodium carbonate + Jaggery + 7up	125 g + 250 g + 250 ml; Bolus followed by 7up	1
14	Potassium nitrate + Potassium bicarbonate + Hukka water	25 g + 25 g + 250 ml; Mix and give PO	1
15	*Trachyspermum ammi* (seeds) + Soap + Common salt + Water	125 g + 125 g + 250 g + 2 L; Decoction given PO	1
16	*Sesamum indicum* (seeds)	500 ml PO	1
17	*Trachyspermum ammi* (seeds) + *Anethum graveolens* (seeds) + Jaggery + White salt	50 g + 50 g + 250 g + 125 g; MGB/PO	1
18	*Trachyspermum ammi* (seeds) + *Vernonia anthelmintica* (seeds) + Jaggery + *Brassica campestris* (seed oil) + Water	50 g + 50 g + 250 g + 250 ml + 250 ml; Decoction PO	1
19	*Vernonia anthelmintica* (seeds) + *Zingiber officinale* (rhizome) + Milk + Water	50 g + 50 g + 250 ml + 250 ml; Grind and give PO	1
	**Total entries**		**22**
	**Dermatitis**		
1	*Brassica campestris* (seed oil)	50-100 ml; Topical application	5
2	Hukka water	Topical application	5
3	*Eruca sativa* (seed oil)	100 ml; Topical application	2
4	*Eruca sativa* (seed oil) + Sulfur	250 ml + 50 g; Topical application	2
6	*Cicer arietinum* (seeds) + *Eruca sativa* (seed oil)	250 g + 250 ml; GMB/PO	1
7	*Azadirachta indica* (leaves) *+* Alum + Common salt + Water	250 g + 25 g + 50 g + 3 L; Decoction applied topically	1
8	*Brassica campestris* (seed oil) + *Eruca sativa* (seed oil)	50 ml + 50 ml; Topical application (massage)	1
9	*Brassica campestris* (seed oil) + Jaggery	50 ml + 50 g; Topical application	1
10	*Brassica campestris* (seed oil) + Yogurt	Topical application	1
13	*Eruca sativa* (seeds) + Common salt	Topical application	1
14	*Sesamum indicum* (seed oil)	50 ml; Topical application	1
	**Total entries**		**21**
	**Diarrhea**		
1	*Allium cepa* (bulb) + Common salt	2 bulbs + 50 g; MGB/PO	1
2	*Citrullus colocynthus* (fruit) + *Vernonia anthelmintica* (seeds) + Black salt + Common salt	50 g + 50 g + 50 g + 50 g; MGB/PO	1
3	Milk + *Brassica campestris* (seed oil)	250 ml + 125 ml; Decoction given PO	1
4	*Prunus dulcis* (seeds) + Jaggery	7-10 seeds + 250 g; MGB/PO	1
5	*Vernonia anthelmintica* (seeds) + Black salt + *Citrillus colocynthus* (fruit)	50 g + 50 g + 50 g; MGB/PO	1
	**Total entries**		**5**
	**External parasite**		
3	*Azadirachta indica* (leaves) + Water	250 g + 1 L; Topical application of decoction	2
4	*Brassica campestris* (seed oil) + Sump oil	125 ml + 125 ml; Topical application	1
5	*Brassica campestris* (seed oil) + Kerosene oil	250 ml + 125 ml; Topical application	1
6	*Curcuma longa* (rhizome)	Topical application of powder	1
7	*Eruca sativa* (seeds) + *Triticum aestivum* (flour)	50 g + 250 g; MGB/PO	1
9	*Brassica campestris* (seeds)	Topical application of ground seeds	1
	**Total entries**		**11**
	**Eye problem**		
	**Total entries**		**2**
	**Fever**		
1	*Vernonia anthelmintica* (seeds) *+ Anethum graveolens* (seeds) *+* Black salt + *Zingiber officinale* (rhizome) *+ Withania coagulans* (leaves) + Common salt + *Picrorhiza kurroa* (rhizome) *+ Citrullus colocynthis* (fruit) + Jaggery + *Allium cepa* (bulb) *+ Capsicum annum* (fruit)	125 g + 125 g + 12 g + 50 g + 250 g + 50 g + 250 g + 500 g + 250 g + 250 g + 250 g; GMB for 8 days and give in equal doses PO	2
2	*Allium cepa* (bulb) + *Capsicum annum* (fruit) + *Zingiber officinale* (rhizome) + Jaggery + *Allium sativum* (bulb)	125 g + 50 g + 25 g + 125 g + 25 g; GMB/PO	1
3	Black salt + *Trachyspermum ammi* (seeds)	125 g + 50 g PO	1
4	Common salt + *Trachyspermum ammi* (seeds) + Water	50 g + 50 g + 50 ml; MGB/PO	1
7	Jaggery + *Amomum subulatum* (fruit)	250 g + 50 g; MGB/PO	1
9	*Piper nigrum* (pepper corn) *+ Trachyspermum ammi* (seeds) *+* Water + Sugar	500 g + 100 g + 500 ml + 1 kg; Mix, fry and make custard – 250 g daily for 5 days	1
	**Total entries**		**10**
	**Haematuria**		
1	Butter + *Piper nigrum* (pepper corn)	125 g + 25 g; MGB/PO	1
2	*Zea mays* (flour) + Brown sugar + Water	500 g + 500 g + 2 L PO	1
3	Potassium bicarbonate + Potassium nitrate	50 g + 50 g PO	1
	**Total entries**		**4**
	**Heat stress**		
1	*Trachyspermum ammi* (seeds) *+* Common salt	50 g + 125 g; Soak seeds overnight, grind and mix with salt – give PO	2
2	*Allium cepa* (bulb) + Jaggery	250 g + 250 g; Half boil bulbs, mix in jiggery and give PO	1
3	Dalda ghee + *Vernonia anthelmintica* (seeds)	125 g + 50 g; MGB/PO	1
4	*Trachyspermum ammi* (seeds) *+* Jaggery	250 g + 250 g PO	1
5	*Vernonia anthelmintica* (seeds) + Sugar	50 g + 125 g; MGB/PO	1
6	Water	As much as animal can drink	1
7	*Zingiber officinale* (rhizome) *+ Halorrhena pubescens* (bark) + Black salt + Common salt + Water	50 g + 50 g + 25 g + 25 g + 1 L; Soak all in earthen utensil for 24 hrs; PO	1
8	*Hordeum vulgare* (seeds) + Water	2 kg + 4 L; Soak overnight; PO	1
	**Total entries**		**9**
	**Indigestion**		
1	*Capsicum annum* (fruit) *+ Allium cepa* (bulb) *+ Vernonia anthelmintica* (seeds) *+ Trachyspermum ammi* (seeds) *+ Zingiber officinale* (rhizome) + Jaggery + *Citrullus colocynthis* (fruit) + Black salt + Common salt	50 g + 250 g + 50 g + 50 g + 50 g + 100 g + 100 g + 100 g + 100 g; MGB/PO	1
2	*Citrullus colocynthis* (fruit) *+ Picrorhiza kurroa* (rhizome) *+ Vernonia anthelmintica* (seeds) *+* Black salt + Ammonium chloride	100 g + 10 g + 10 g + 50 g + 3 balls; Mixed in hot water and given PO	1
3	Jaggery + *Trachyspermum ammi* (seeds) *+ Zingiber officinale* (rhizome) *+ Allium cepa* (bulb) *+ Allium sativum* (bulb)	250 g + 50 g + 50 g + 250 g + 50 g; PO	1
4	*Vernonia anthelmintica* (seeds) *+ Anethum graveolens* (seeds) *+* Black salt + *Zingiber officinale* (rhizome) *+ Withania coagulans* (leaves) *+* Common salt + *Picrorhiza kurroa* (rhizome) *+ Citrullus colocynthis* (fruit) *+* Jaggery + *Allium cepa* (bulb) *+ Capsicum annum* (fruit)	125 g + 125 g + 12 g + 50 g + 250 g + 50 g + 250 g + 500 g + 250 g + 250 g + 250 g; MGB/PO, equally divided in 8 balls and given one daily	1
5	*Zingiber officinale* (rhizome) *+ Trachyspermum ammi* (seeds) *+ Foeniculum vulgare* (seeds) + Black salt + Jaggery	50 g + 50 g + 50 g + 50 g + 250 g; MGB/PO	1
	**Total entries**		**5**
	**Internal parasites**		
1	*Mollotus philipinensis* (fruit) + Jaggery	50 g + 250 g PO	6
2	*Mallotus philippinensis* (fruit) + Yogurt	10 g + 125 g PO	3
3	*Acacia nilotica (branches)* + Jaggery	125 g + 125 g PO	1
4	Jaggery + *Azadirachta indica* (leaves)	250 g + 500 g; Jaggery 10 minutes before *A. indica* leaves PO	1
5	Jaggery + *Oryza sativa* (whole plant)	250 g + 1 kg; Give Jaggery on first day and *O. sativa* next day	1
6	Jaggery + Yogurt + *Mollotus philpinensis* (fruit)	125 g + 250 g + 50 g PO	1
7	*Lagenaria siceraria* (leaves)	250 g; Ground and given PO	1
8	*Linum usitatissimum* (seeds) *+ Lepidium sativum* (seeds) *+* Jaggery + *Mallotus philippinensis* (fruit)	60 g + 60g + 250 g + 25 g; PO	1
9	*Mallotus philippinensis* (fruit) + Milk whey	10 g + 250 ml PO	1
10	Nurru (stem) + Jaggery	250 g + 250 g; Given jaggery first and then nurru	1
11	*Ricinus cummunis* (flower oil)	250 ml oil PO	1
12	*Ricinus cummunis* (flower oil)	250 ml PO	1
	**Total entries**		**31**
	**Lameness**		
1	*Brassica campestris* (seed oil)	50-100 ml; Topical application	8
2	*Sesamum indicum* (seed oil)	50 ml; Topical application	4
3	Copper sulfate + Jaggery	10 g + 100 g PO	3
5	*Citrus limon* (fruit)	Half piece for massage on the affected site	2
7	*Brassica campestris* (seed oil) + Kerosene oil	50 ml + 50 ml; Topical application	1
12	*Azadirachta indica* (leaves) + Common salt + Water	250 g + 250 g + 6 Lit; Topical application of decoction	1
13	*Azadirachta indica* (leaves) + Water	100 g + 1 L; Topical application of decoction	1
14	*Azadirachta indica* (leaves) + Water + Common salt + Alum	250 g + 4 L + 1 kg + 250 g; Topical application of decoction	1
15	*Brassica campestris* (seed oil) + Kerosene oil + *Capsicum annum* (fruit)	50 ml + 50 ml + 25 g; Topical application	1
16	*Brassica campestris* (seed oil)	Topical application of semi-hot oil	1
17	*Cascuta reflexa* (whole plant) + Ghee	*C. reflexa* fried in oil; Topical application	1
18	*Cicer arietinum* (seeds)	50 g grains tied on affected area by putting in a cloth bag	1
19	*Cicer arietinum* (seeds) + Jute bag	250 g grains tied on affected area by putting in a jute bag	1
21	Common salt + *Triticum aestivum* (flour) + Water	Topical application of hot mixture	1
22	*Geranium wallichianum* (seeds) *+ Brassica campestris* (seed oil)	50 g + 50 ml; Mix, fry and apply topically	1
23	*Geranium wallichianum* (seeds) *+ Brassica campestris* (seed oil)	50 g + 50 ml; Mix, fry and apply topically	1
24	Jaggery + *Curcuma longa* (rhizome)	250 g + 50 g; MGB/PO	1
27	*Medicago sativa* (leaves) + Common salt	500 g + 100 g; Mix and apply on the affected area for 2-3 days	1
28	*Mangifera indica* (fruit)	Topical application on the lesion	1
29	*Myristica fragrans* (fruit) *+ Geranium wallichianum* (seeds) *+ Eugenia caryophyllata* (fruit) *+ Sesamum indicum (seeds oil) + Brassica campestris* (seed oil)	50 g + 50 g + 50 g + 250 ml + 250 ml; Topical application	1
30	*Olea europaea* (fruit) *+ Cocus nucifera (oil)* + Fish + Sump oil	250 ml + 250 ml + 250 ml + 250 ml; Topical application	1
31	*Pennisetum glaucum (whole)* + Jaggery	125 g + 125 g; MGB/PO	1
32	*Peganum harmala* (fruit) + Alum + Jaggery	Topical application	1
34	*Sesamum indicum* (seeds) *+ Eugenia caryophyllata* (fruit) *+ Myristica fragrans* (fruit)	125 ml + 10 g + 50 g; Topical hot application	1
35	*Trachyspermum ammi* (seeds) *+ Anethum graveolens* (seeds) *+ Vernonia anthelmintica* (seeds) + Jaggery	125 g + 125 g + 50 g + 500 g; MGB/PO	1
36	*Vernonia anthelmintica* (seeds) *+ Anethum graveolens* (seeds)	250 g + 250 g; 50 g daily	1
37	Water + *Triticum aestivum* (flour) + Common salt + Alum	2 L + 500 g + 125 g + 25 g; Topical application of decoction	1
38	*Zingiber officinale* (rhizome) *+ Picrorhiza kurroa* (rhizome) *+ Vernonia anthelmintica* (seeds) *+ Grewia asiatica* (fruit) + Jaggery	250 g + 250 g + 250 g + 250 g + 250 g; GMB/PO	1
39	*Ricinus cummunis* (bark) *+ Sesamum indicum* (seeds oil)	250 g + 250 ml; Boiled bark in seed oil and applied on affected part for 2-3 days	1
	**Total entries**		**58**
	**Pain associated with infection**		
1	*Sesamum indicum* (seed oil) *+ Eugenia caryophyllata* (fruit) *+ Myristica fragrans* (fruit)	125 ml + 25 g + 25 g; Mixed, fried and applied topically	1
2	Sodium carbonate + Jaggery	One spoon + 250 g; MGB/PO	1
3	*Nicotiana tabacum* (leaves) + Jaggery	50 g + 250 g; MGB/PO	1
4	*Zingiber officinale* (rhizome) + *Allium cepa* (bulb) *+ Glycyrrhiza glabra* (roots) *+ Capsicum annum* (fruit) *+* Water	1 kg + 3 kg + 1 kg + 1 kg + 1 litre; MGB/PO	1
5	*Alum + Peganum harmala* (fruit) *+ Jaggery*	50 g + 125 g + 125 g; Mix, fry and give PO one spoon a day	1
	**Total entries**		**5**
	**Quidding**		
1	*Capsicum annum* (fruit) *+ Jaggery* + Common salt + *Allium cepa* (bulb)	50 g + 250 g + 50 g + 250 g; MGB/PO	1
	**Total entries**		**1**
	**Retention of urine**		
1	*Capsicum annum* (fruit)	Applied *Capsicum annum* (fruit) L. on urethral opening	2
2	*Brassica campestris* (seed oil) + Milk	125 ml + 250 ml; PO	1
3	*Brassica campestris* (seed oil) + Water	250 ml + 250 ml; given decoction PO	1
7	*Trachyspermum ammi* (seeds)	125 g seeds soaked overnight in water and given PO	1
	**Total entries**		**8**
	**Swelling**		
1	*Acacia nilotica (branches) + Ficus religiosa* (bark) *+* Water + *Brassica campestris* (seed oil)	250 g + 250 g + 2 L + 25 ml; Topical application of decoction	1
2	*Azadirachta indica* (leaves) + Alum + Common salt + Water	250 g + 50 g + 1 spoon + 2 L; Topical application of decoction	1
3	Leather + *Brassica campestris* (seed oil)	500 g + 500 ml; Heated leather in seed oil and applied on inflammation	1
4	*Peganum harmala* (fruit)	125 g PO	1
	**Total entries**		**4**
	**Tetanus**		
1	Egg + *Zingiber officinale* (rhizome) *+ Myristica fragrans* (fruit) *+ Picrorhiza kurroa* (rhizome)	One + 25 g + One seed + 25 g; MGB/PO – 3 days	1
2	*Myristica fragrans* (fruit) + *Picrorhiza kurroa* (rhizome) + Jaggery	One fruit + 25 g + 10 g; MGB/PO and cuts on nose for bleeding	1
	**Total entries**		**2**
	**Toxemia**		
1	*Capsicum annum* (fruit) + Common salt	250 g + 125 g; PO	1
2	*Capsicum annum* (fruit) + Ghee + Water	250 g + 250 g + 1000 ml; decoction PO	1
3	*Capsicum annum* (fruit) + Ghee	250 g + 250 g; Ground, fried and given PO	1
4	*Capsicum anuum* (fruit) *+ Trigonella foenum-graecum* (seeds) *+ Vernonia anthelmintica* (seeds) *+ Anethum graveolens* (seeds) *+* Water	1000 g + 500 g + 250 g + 250 g + 10 litre; Boiled and made custard – 200 g daily	1
5	*Piper nigrum* (pepper corn) + Ghee	125 g + 125 g; Ground, mixed in warm ghee and given PO	1
6	*Vernonia anthelmintica* (seeds) *+ Anethum graveolens* (seeds) *+ Trigonella foenum-graecum* (seeds) *+ Capsicum anuum* (fruit) + Water	250 g + 500 g + 500 g + 500 g + 5 litre; Decoction PO	1
	**Total entries**		**6**
	**Weakness**		
1	*Vernonia anthelmintica* (seeds) *+ Anethum graveolens* (seeds) *+* Black salt + *Zingiber officinale* (rhizome) *+ Withania coagulans* (leaves) *+* Common salt + *Picrorhiza kurroa* (rhizome) *+ Citrullus colocynthis* (fruit) + Jaggery + *Allium cepa* (bulb) *+ Capsicum annum* (fruit)	125 g + 125 g + 12 g + 50 g + 250 g + 50 g + 250 g + 500 g + 250 g + 250 g + 250 g; MGB/PO – 4 boluses for 4 days	1
2	*Allium cepa* (bulb) + Jaggery	250 g + 250 g; MGB/PO	1
3	*Brassica campestris* (seed oil) *+ Triticum aestivum* (flour)	125 ml + 25 g PO	1
4	*Calotropis procera* (buds) *+ Brassica campestris* (seed oil)	100 g + 100 ml; Decoction PO	1
*5*	Common salt + *Capsicum annum* (fruit) *+ Picrorhiza kurroa* (rhizome) *+ Citrullus colocynthis* (fruit)	125 g + 125 g + 10 g 250 g; Decoction PO	1
6	*Zea mays* (flour) + Brown sugar + Water	250 g + 250 g + 10 L PO	1
7	*Lepidium sativum* (seeds) + Jaggey + Water + Milk	50 g + 50 g + 100 ml + 2000 ml; Made custard and given PO	1
8	*Vernonia anthelmintica* (seeds) *+ Foeniculum vulgare* (seeds) *+* Jaggery	50 g + 50 g + 125 g; MGB/PO	1
	**Total entries**		**13**
	**Wound**		
1	*Brassica campestris* (seed oil)	50-100 ml; Topical application	25
3	*Brassica campestris* (seed oil) *+ Lawsonia inermis* (leaves)	50 ml + 50 g; Applied oil first and then leaves of *L. inermis* on wound	5
4	*Curcuma longa* (rhizome) *+ Brassica campestris* (seed oil)	250 g + 250 ml; Topical application	4
5	*Lawsonia inermis* (leaves)	Topical application	4
6	*Lawsonia inermis* (leaves) + Water	50 g + 50 ml; Topical application	4
7	*Azadirachta indica* (leaves) + Water	250 g + 500 ml; Washed wounds with hot decoction	3
8	*Brassica campestris* (seed oil) + Kerosene oil	150 ml + 150 ml; Topical application	3
9	*Lawsonia inermis* (leaves) *+ Brassica campestris* (seed oil)	250 g + 25 ml; Topical application	2
10	*Azadirachta indica* (leaves) *+* Common salt + Water	500 g + 250 g + 2 Litre; Washed wounds with decoction	2
11	*Brassica campestris* (seed oil) + Carbon of cell	Topical application	2
12	*Brassica campestris* (seed oil) *+* Water	50 ml + 500 ml; Washed wound with decoction and applied oil	2
15	Dalda ghee + *Curcuma longa* (rhizome)	25 g + 10 g; Applied mild hot ghee on wound and sprinkled *C. longa* powder	2
16	Hot iron + *Brassica campestris* (seed oil)	Topical application	1
17	*Azadirachta indica* (leaves) *+ Brassica campestris* (seed oil)	125 g + 125 ml; Washed the wounds with decoction and poured oil on the wound	1
18	*Azadirachta indica* (leaves) *+ Brassica campestris* (seed oil) *+* Common salt	500 g + 500 ml + 100 g; Boiled leaves in seed oil and salt and applied on wound for 2-3 days	1
19	*Azadirachta indica* (leaves) *+ Brassica campestris* (seed oil) *+* Alum	500 g + 500 ml + 100 g; Boiled leaves in oil and alum, washed wounds with extract	1
20	*Azadirachta indica* (leaves) *+ Curcuma longa* (rhizome) *+* Alum + Milk fat	250 g + leaves + 50 g + 25 g + 50 g; all ingredients were ground to powder, Then applied milk fat on wound then powder	1
21	*Azadirachta indica* (leaves) + water + Dettol	250 g + 2 litre + few drops; Mixed and washed the wound	1
22	*Withania somnifera* (leaves) + *Brassica campestris* (seed oil)	1 kg + 500 ml; Boiled leaves in oil and tied on wounds for 3 days	1
23	*Zea mays* (flour) + Common salt + Water	250 g + 250 g + 250 ml; Topical application	1
24	*Lens culinaris* (seeds) *+ Brassica campestris* (seed oil)	250 g + 250 ml; Topical application of decoction	1
25	*Brassica campestris* (seed oil) + Kerosene oil + *Curcuma longa* (rhizome)	50 ml + 50 ml + 100 g; Topical application	1
26	*Brassica campestris* (seed oil) *+* Soap	125 ml; Washed wound with soap and applied oil	1
27	*Trifolium alexandrinum* (whole)	Put *Trifolium alexandrinum* in closed utensils for hours and after that tied on wound	2
28	*Capsicum anuum* (fruit)	50 g; Topical application of powder	1
29	*Cicer arietinum* (seeds)	50 g; wraped cloth and tied on wound, kept on pouring water on cloth	1
33	*Citrus limon* (fruit)	Cut into half and rubbed on wound	1
31	*Curcuma longa* (rhizome)	10 g; Topical application	1
32	*Eruca sativa* (seeds) *+ Brassica campestris* (seeds)	Topical application	1
33	*Geranium wallichianum* (seeds) *+ Brassica campestris* (seed oil)	50 g + 250 ml; Topical application	1
34	Ghee *+ Curcuma longa* (rhizome)	25 g + 25 g; Mixed, fried and applied topically	1
35	*Lawsonia inermis* (leaves) + Alum	50 g + 50 g; Topical application	1
36	*Lawsonia inermis* (leaves) + Alum + *Brassica campestris* (seed oil)	125 g + 50 g + 50 ml; Topical application	1
37	*Lawsonia inermis* (leaves) + Alum + Water	250 g + 50 g + 200 ml; Heated the alum, ground and mixed with water and *L. inermis*; Topical application	1
38	Leather + *Brassica campestris* (seed oil)	Burnt leather to ash, mixed with brassica oil and applied on wound	1
39	*Vernonia anthelmintica* (seeds) + *Triticum aestivum* (flour)	125 g + 125 g PO	1
40	*Ziziphus jujube* (leaves) *+ Brassica campestris* (seed oil)	250 g + 250 ml; Boiled leaves in seed oil and tied on wounds for 2-3 days	1

**Table 4:** Frequency of plants used for the treatment of equine diseases/ conditions documented by local veterinary healers from three districts (Faisalabad, Sargodha and Lahore) of Punjab, Pakistan

**Conditions**	**Plants**	**Entries**	**Prescriptions/**	**Plants as part of prescriptions for the same disease more than one time**^**[1]**^
			**remedies**	
Anorexia	17	23	19	*Allium (A.) cepa* (12), *Capsicum (Cp.) annum* (9), *Zingiber (Z.) officinale* (8), *Vernonia (V.) anthelmintica* (6), *Citrullus (Ct.) colocynthis* (4), *Trachyspermum (T.) ammi* (4), *Picrorhiza (Pr.) kurroa* (3), *Piper (P.) nigrum* (3), *Zea mays* (2)
Bad habits	1	5	2	***-***
Bronchitis	12	33	21	*Glycyrrhiza glabra* (11), *A. cepa* (8), *Rosa damascena* (5), *Z. officinale* (4), *P. nigrum* (2), *T. ammi* (2), *V. anthelmintica* (2)
Colic	9	24	21	*Brassica (B.) campestris* (4), *T. ammi* (3), *A. cepa* (2), *Nicotiana tabacum* (2), *V. anthelmintica* (2)
Dermatitis	6	32	16	*Eruca sativa* (5), *B. campestris* (4)
Diarrhoea	5	5	5	*Ct. colocynthus* (2), *V. anthenthelmintica* (2)
Ectoparasites	5	23	11	*B. campestris* (2)
Eye problem	**-**	2	2	***-***
Fever	11	12	9	*T. ammi* (3), *A. cepa* (2), *Capsicum (Cp)annum* (2), *Z. officinale* (2)
Haematuria	2	4	4	**-**
Heat stress	6	10	8	*T. ammi* (2), *V. anthelmintica* (2)
Indigestion	11	6	6	*Z. officinale* (4), *A. cepa* (3), *T. ammi* (3), *Cp. annum* (2), *Ct. colocynthis* (2), *Pr. kurroa* (2), *V. anthelmintica* (2)
Endoparasites	8	31	12	*Molottus philpinensis* (4), *Ricinus cummunis* (2)
Lameness	21	58	40	*B. campestris* (7), *Sesamum indicum* (4), *Azadirachta (Az.) indica* (3), *Geranium wallichianum* (3), *V. anthelmintica* (3), *Anethum (An.) graveolens* (2), *Cicer arietinum* (2), *Eugenia caryophllata* (2), *Myristica (M.) fragrans* (2)
Pain	9	5	5	***-***
Quidding	2	3	3	***-***
Urine retention	3	10	8	*B. campestris* (2)
Swelling	5	11	7	*B. campestris* (2)
Tetanus	3	2	2	*M. fragrans* (2), *Pr. kurroa* (2)
Toxemeia	5	7	7	*Cp. annum* (5), *An. graveolens* (2), *Trigonella foenum-graceum* (2), *V. anthelmintica* (2)
Weakness	14	23	13	*A. cepa* (2), *B. campestris* (2), *Cp. annum* (2), *Ct. colocynthis* (2), *Pr. kurroa* (2), *V. anthelmintica* (2)
Wound	16	121	57	*B. campestris* (20), *Az. indica* (7), *Lawsonia inermis* (6), *Curcuma longa* (5)

^[1]^Plants were used in more than one prescription because of difference either in their dose, composition of the prescription, mode of preparation and administration, vehicles, etc.

### Conditions reported against plant usage

Number and nature of TVPs documented for the treatment of different diseases/conditions have been summarized in the Table 2. It is evident from the data (Table 4) that maximum number of plant based remedies/prescriptions was documented for the treatment of wounds (n = 57) followed by lameness (n = 40), bronchitis and colic (n = 21), anorexia (n = 19), dermatitis (n = 16), weakness (n = 13), internal parasites (n = 12), external parasites (n = 11), fever (n = 09), heat stress and retention of urine (n = 08), swelling and toxemia (n = 07), indigestion (n = 06), diarrhoea and pain (n = 05), haematuria (n = 04), quidding (n = 03), bad habits, eye problem and tetanus (n = 02). Similar trend was seen for the number of TVPs used for different diseases/conditions being highest (n = 121) for wounds and the lowest for tetanus (n = 02). Maximum number of plants were used against lameness (n = 21) followed by anorexia (n = 17), wounds (n = 16), weakness (n = 14), bronchitis (n = 12), etc. as shown in the Table 4.

### Approaches adapted in plant usage

Seeds were the most frequently used (n = 16/60) part of plants as such or as their oils followed by leaves (n = 12/60) and fruit (n = 11/60). The other parts of plants used were: whole plant, rhizome, bark, branches, bulb, buds, flour, pepper corn, roots, etc (Table 2). Prescriptions for treatment/control of different conditions of equines were based on single or multiple plants. Most of the recipes were prepared by mixing and grinding the ingredients. The powder was then made into physic balls as bolus to be given orally or decoctions were prepared for drenching the animals and/or used for topical application as washing, spraying, ointment, liniment, massage, etc. The prescriptions also differed in dose, method of preparation, and mode of administration of plants and/or materials other than plants; within and amongst the diseases/conditions. In some cases, frying, burning (to create smoke around animals), pouring, drinking, soaking before use, forced inhalation and steaming of ingredients were practiced.

## Discussion

There are several recent evidences of plant based treatment and control strategies from Pakistan, especially for parasitism [11,12]. This has been supported by repellent activity of *Moringa oleifera*[17], an indication to be used against ecto-parasites. Plants from different geographical regions have produced variable results [18] as the synthesis of secondary plant products can be affected by environmental/growing conditions. Steroid saponins show pharmacologic actions like antifungal, antibacterial, anti-inflammatory and hypocholesteremic influences (Wang *et al*., [19]). So, plants producing saponins and organosulfur compounds like those of genus *Allium* can be used in the conditions described above.

Large number of qualified veterinarians also advocates the use of phytotherapy, other than preventive medication, but these practices are less organized in the form of scientific reports and are usually transferred orally as these have been developed by farmers, rather than by scientists in sophisticated laboratories. This situation is typical of a rural underdeveloped culture like that of pastoralists of Africa [20] and other parts of world having dependence on phytotherapy for their animals. Inadequate access to modern health care facilities due to cost-ineffectiveness, inherited beliefs, empirical evidence of efficacy, cultural acceptability and availability of botanicals at the farmers’ doorstep are the main factors [21] that lead to dependence of livestock farmers on the phytotherapy. The indigenous knowledge and skills can contribute towards development of phytotherapy in less developed areas of the world [22,23]. Plants are considered to possess relatively higher bioactive secondary compounds, thus hold promise for drug discovery. Most of the plant-derived chemicals are secondary metabolites, of which at least 12,000 have been isolated; a number estimated to be less than 10% of the total [24]. Nok *et al.*[25,26] and Nok and Williams [27] have discussed the active principles as well as the mechanisms of action of some plant extracts that are used in phytotherapy.

Fruitful efforts have also been made previously to document TVPs in some parts of Punjab, Pakistan focusing livestock but not including equines [28]–[32]. Equine industry has the ready and largely uninformed access to herbal products. Therefore, at least to the extent of equines, herbals are more than traditional veterinary medicine. The researchers have, therefore, focused on documentation and validation of usage of plants based on the claims of traditional healers [33]–[37].

Results of the present study have revealed that equine owners and/or traditional veterinary healers have great wealth of indigenous knowledge based on their practices and experience, which is evident from the number of plants (n = 60) used for treatment of different diseases/conditions of equines in Faisalabad, Sargodha and Lahore. Use of plants in multiple disorders indicates diversity of their pharmacological and toxicological impacts [38]. Plants have more than one mode of actions; therefore, provide broad spectrum activities in different diseases [39] due to diversity of phyto − chemicals. Twenty − four plants including *Anethum graveolens, Bambusa bambos, Cascuta reflexa, Citrus limon, Cocus nucifera, Ficus religiosa, Geranium wallichianum, Grewia asiatica, Halorrhena pubescens, Lagenaria siceraria, Lepidium sativum, Mangifera indica, Medicago sativa, Myristica fragrans, Nigella sativa, Oryza sativa, Peganum harmala, Pennisetum glaucum, Picrorhiza kurroa, Piper betle, Prunus dulcis, Trifolium alexandrinum, Withania somnifera* and *Zea mays* were found to be used for different indications in equines. As far as could be ascertained, there is no published literature on the use of plants for the treatment of ailments in equines. There are thousands (≈250,000) of species of plants naturally available [40], with a low proportionate exploited for medicinal purposes. Further, 5–5% of the higher plants have been investigated for their active constituents against a wide range of infectious and non-infectious diseases of humans and animals (Pieters and Vlietinck [41]). Plants are primary source of natural products used by traditional healers in 80% of the developing societies [42]. There was about 40% repetition in the ethno-botanical preparations (EBPs) documented in the present study and those documented by others for other species of animals [28]–[32]). It has been reported previously that different parts of the same plant (leaves, fruits, flowers, seeds, seed kernels, latex, stem, grains, bulbs, tuber, roots, basal rosette, bark, thallus, shoots, wood, buds, aerial parts, branches, etc.) and variety of solvents used for their extraction diversify their usage ([43]–[46]). Variation in the doses and mode of preparation of remedies within and among different conditions has also been reported elsewhere [11,28,30,32,47]. The aspect of non-standardized doses in phytotherapy have been criticized, because of toxicity constraints, under dosing, and cost; however, cost can be reduced by proper standardization of doses [48,49]. The common adage that natural is synonymous with safe, has led to significant and widespread disease [50], and it is critical that those involved in equine husbandry and health care are aware of the potential dangers of herbal medicine. Therefore, researches on the standardization of doses regarding efficacy *vs* safety should be carried out.

## Conclusions

The current research suggests that EBPs have a crucial role in animal health and production in the study area. The current study revealed a diverse range of plants which is in practice to treat the prevalent ailments in equine population of Punjab. The pitfalls of TVPs found in this study were related with the improper diagnosis of diseases (some cases), non-standardization of dosages, mode of preparation and administration, and lack of understanding regarding importance of value addition (e.g., validation) to the existing practices, adverse/overdosing effects and documentation of indigenous knowledge. For example, traditional healers were not aware of the minimum essentials of parasite biology and strategic worm control practices. Fundamental issues in phytotherapy; however, are the dose, efficacy and safety left to an educated guess or is completely ignored. However, a handsome volume of the indigenous knowledge has been documented for the first time in the region in relation to the treatment of equines which provides a baseline for future scientific investigations in phytochemistry. The promising candidates of plant origin can be isolated through modern chemistry protocols and authenticated for their medical value after *in vivo* and *in vitro* experimentations.

## Competing interests

We declare that none of the authors have competing interests.

## Authors’ contributions

ZI: designed the project for the Ph.D. research of KG who was actively involved in the field surveys and compilation of the data. MS provided the transport and consultation services for approaching the equines of the study area. MSS provided comments and suggestions during drafting and reporting of the data and wrote the draft of manuscript. Q was involved in writing and reviewing of manuscript. All authors approve the final submission of the manuscript.

## References

[B1] Urquhart GM Armour J Duncan JL Dunn AM Jennings FW Veterinary Parasitology 2007 2 UK: Blackwell Science

[B2] Goraya K Iqbal Z Sajid MS Muhammad G Frequency distribution of equine diseases in three metropolises of the upper Punjab, Pakistan Int J Agric Biol 2013 00 000 000

[B3] Lans C Turner N Brauer G Lourenco G Georges K Ethnoveterinary medicines used for horses in Trinidad and in British Columbia, Canada J Ethnobiol Ethnomed 2006 2 31 10.1186/1746-4269-2-31 16893454 PMC1559680

[B4] Cox PA Will tribal knowledge survive the millennium? Science 2000 287 44 45 10.1126/science.287.5450.44 10644221

[B5] Ole−Miaron JO Ethnoveterinary practices of the Loitokitock Maasai: impact on the environment Vet J 1997 17 159 167

[B6] Lans C Creole remedies: case studies of ethnoveterinary medicine in Trinidad and Tobago. PhD Thesis 2001 Netherlands: Wageningen University

[B7] Akhtar MS Iqbal Z Khan MN Lateef M Anthelmintic activity of medicinal plants with particular reference to their use in animals in the Indo-Pakistan subcontinent Small Rumin Res 2000 38 99 107 10.1016/S0921-4488(00)00163-2

[B8] Iqbal Z Lateef M Jabbar A Muhammad G Khan MN Anthelmintic activity of *Calotropis procera* (Ait.) Ait. F. flowers in sheep J Ethnopharmacol 2005 102 256 261 10.1016/j.jep.2005.06.022 16085379

[B9] Iqbal Z Sarwar M Jabbar A Ahmad S Nisa M Sajid MS Khan MN Mufti KA Yaseen M Direct and indirect anthelmintic effects of condensed tannins in sheep Vet Parasitol 2007 144 125 131 10.1016/j.vetpar.2006.09.035 17097807

[B10] Lateef M Iqbal Z Sajid MS Abbas RZ Sindhu ZUD Akhtar M Khan MN Awais MM Iqbal A Ain QU An account of botanical anthelmintics and methods used for their evaluation Rev Vet Anim Sci 2013 1 7 14

[B11] Sindhu ZUD Iqbal Z Khan MN Jonsson NN Siddique M Documentation of ethno − veterinary practices used for treatment of different ailments in selected a hilly area of Pakistan Int J Agric Biol 2010 12 353 358

[B12] Sindhu ZUD Shafiq-Ullah Abbas RZ Iqbal Z Hameed M Inventory of ethno-veterinary practices used for the control of parasitic infections in district Jhang, Pakistan Int J Agric Biol 2012 14 922 928

[B13] Anonymous Pakistan livestock census 2006 Pakistan, Islamabad: Agricultural Census Organization, Ministry of Economic Affairs and Statistics

[B14] Dunn T Rapid rural appraisal: a description of the methodology and its application in teaching and research at Charles Stuart University 1992–1994 Wagga Wagga Australia: Rural Society

[B15] Thrusfield M Veterinary epidemiology 2007 3 Blackwell science 231 232

[B16] Etkins NL Anthropological methods in ethnopharmacology J Ethnopharmacol 1993 38 93 104 10.1016/0378-8741(93)90004-O 8510473

[B17] Ashfaq M Ashfaq U Evalauation of mosquitocidal activity of water extract of *Moringa Oleifera* seeds against *Culex Quinuefasciatus* (Diptera: Culicidae) in Pakistan Pak Entomol 2012 34 21 26

[B18] Waller PJ Bernes G Thamsborg SM Sukura A Richter SH Ingebrigtsen K Hoglund J Plants as de-worming agents of livestock in the Nordic countries: historical perspective, popular beliefs and prospects for the future Acta Vet Scand 2001 42 31 44 10.1186/1751-0147-42-31 11455900 PMC2202332

[B19] Wang P Su Z Yuan W Deng G Li S Phytochemical constituents and pharmacological activities of Eryngium L. (Apiaceae) Pharmaceutical Crops 2012 3 99 120 10.2174/2210290601203010099

[B20] De Leeuw PN McDermott JJ Lebbie SHB Monitoring of livestock health and production in sub − Saharan Africa Prev Vet Med 1995 25 195 212 10.1016/0167-5877(95)00547-1

[B21] Bennet−Jenkins E Bryant C Novel sources of anthelmintics Int J Parasitol 1996 26 937 947 10.1016/S0020-7519(96)80068-3 8923141

[B22] Brokensha DW Warren DM Werner O Indigenous knowledge systems and development 1980 Lanham, MD: University Press of America

[B23] IDS Whose knowledge counts? IDS Bull 1979 10 2

[B24] Schultes RE The kingdom of plants: medicines from the earth, Thomson, WAR 1978 New York, NY: McGraw−Hill Book Co 208

[B25] Nok AJ Esievo AN Longet I Arowosafe S Onyenekwe PC Gimba CE Kagbu JA Trypanocidal potentials of *Azadirachta indica*: *in vivo* activity of leaf extract against *Trypanosoma brucei* J Clin Biochem Nutr 1993 15 113 118 10.3164/jcbn.15.113

[B26] Nok AJ Esievo KAN Armbrose A Isaac AI Emmanuel GC Solomon MO James KA Trypanocidal activity of an organotin compound (tri-n-butyltin oxide) toward *Trypanosoma brucei* J Clin Biochem Nutr 1992 13 81 85 10.3164/jcbn.13.81

[B27] Nok AJ Williams S *Allium sativum* induced death of African Trypanosomes Parasitol Res 1996 82 634 637 10.1007/s004360050177 8875572

[B28] Dilshad SMR Rehman N Iqbal Z Muhammad G Iqbal A Ahmed N An inventory of the ethnoveterinary practices for reproductive disorders in cattle and buffaloes, Sargodha district of Pakistan J Ethnopharmacol 2008 117 393 402 10.1016/j.jep.2008.02.011 18384987

[B29] Farooq Z Iqbal Z Mushtaq S Muhammad G Iqbal MZ Arshad M Ethnoveterinary practices for the treatment of parasitic diseases in livestock in Cholistan desert (Pakistan) J Ethnopharmacol 2008 118 213 219 10.1016/j.jep.2008.03.015 18524514

[B30] Jabbar A Iqbal Z Khan MN *In vitro* anthelmintic activity of *Trachyspermum ammi* seeds Phcog Mag2006b 2 126 129

[B31] Khan MK Sajid MS Khan MN Iqbal Z Iqbal MU Bovine fasciolosis: prevalence, effects of treatment on productivity and cost benefit analysis in five districts of Punjab, Pakistan Res Vet Sci 2009 87 70 75 10.1016/j.rvsc.2008.12.013 19181352

[B32] Muhammad G Khan MZ Hussain MH Iqbal Z Iqbal M Athar M Ethnoveterinary practices of owners of pneumatic-cart pulling camels in Faisalabad city (Pakistan) J Ethnopharmacol 2005 97 241 246 10.1016/j.jep.2004.11.008 15707760

[B33] Aqel MB Relaxant effect of the volatile oil of rosmarinus officinalis on tracheal smooth muscle J Ethnopharmacol 1991 33 57 62 10.1016/0378-8741(91)90161-6 1943174

[B34] Lanhers MC Fleurentin J Mortier F Vinche A Younos C Anti-Inflammatory and analgesic effects of an aqueous extract of Harpagophytum procumbens Planta Med 1992 58 17 123 10.1055/s-2006-961411 1529021

[B35] Pearson W Ethnoveterinary medicine: the science of botanicals in equine health and disease Proceedings of the 2nd European equine health and nutrition congress, lelystad 2003 The Netherlands 31 40

[B36] Sommer H Felbinger U Pütz R Reutershan R Schaefer J The effects of an herb mixture on horses with respiratory disease Tierarztl Umsch 1986 41 846 848

[B37] Wagner I Greim C Laufer S Heide L Gleiter CH Influence of willow bark extract cyclooxygenase activity and on tumor necrosis factor alpha or interleukin 1 beta release in vitro and ex vivo Clin Pharmacol Ther 2003 73 272 274 10.1067/mcp.2003.32 12621392

[B38] Iwu M Handbook of African medicinal plants 1993 Boca Raton, FL: CRC Press

[B39] Reichling J Saller R Herbal remedies in veterinary phytotherapy Schweiz Arch Tierheilkd 2001 43 395 403 11525096

[B40] Borris RP Natural products research: perspectives from a major pharmaceutical company J Ethnopharmacol 1996 51 29 38 10.1016/0378-8741(95)01347-4 9213624

[B41] Pieters L Vlietnick AJ Bioguide isolation of pharmacologically active plant components, still a valuable strategy for the finding of new lead compounds Journal of Ethnopharmacology 2005 100 57 60 10.1016/j.jep.2005.05.029 15996842

[B42] Farnsworth NR Akerele O Bingel AS Soejarto DD Guo Z Medicinal plants in therapy Bull World Health Organ 1985 63 965 981 3879679 PMC2536466

[B43] Giday M Asfaw Z Elmqvist T Woldu Z An ethnobotanical study of medicinal plants used by the Zay people in Ethiopia J Ethnopharmacol 2003 85 43 52 10.1016/S0378-8741(02)00359-8 12576201

[B44] Nfi AN Mbanya JN Ndi C Kameni A Vabi M Pingpoh D Yonkeu S Moussa C Ethnoveterinary medicine in the Northern Provinces of Cameroon Vet Res Commun 2001 25 71 76 10.1023/A:1026766219786 11214674

[B45] Ole−Miaron JO The Maasai ethnodiagnostic skill of livestock diseases: a lead to traditional bioprospecting J Ethnopharmacol 2003 84 79 83 10.1016/S0378-8741(02)00283-0 12499079

[B46] Viegi L Pieroni A Guarrera PM Vangelisti R A review of plants used in folk veterinarymedicine in Italy as basis for a databank J Ethnopharmacol 2003 89 221 244 10.1016/j.jep.2003.08.003 14611886

[B47] Deeba F Muhammad G Iqbal Z Hussain I Appraisal of ethno-veterinary practices used for different ailments in dairy animals in peri-urban areas of Faisalabad (Pakistan) Int J Agric Biol 2009 11 535 541

[B48] Bakhiet AO Adam SEI Therapeutic utility, constitutents and toxicity of some medicinal plants Vet Human Toxicol 1995 37 255 258 7571361

[B49] Longuefosse JL Nossin E Medical ethnobotany survey in Martinique J Ethnopharmacol 1996 53 117 120 10.1016/0378-8741(96)01425-0 8887020

[B50] Pearson W Pyrrolizidine Alkaloids in higher plants: hepatic veno-occlusive disease associated with chronic consumption JNFMF 2000 3 87 96

